# A moderate diet restriction during pregnancy alters the levels of endocannabinoids and endocannabinoid-related lipids in the hypothalamus, hippocampus and olfactory bulb of rat offspring in a sex-specific manner

**DOI:** 10.1371/journal.pone.0174307

**Published:** 2017-03-27

**Authors:** María Teresa Ramírez-López, Mariam Vázquez, Ermelinda Lomazzo, Clementine Hofmann, Rosario Noemi Blanco, Francisco Alén, María Antón, Juan Decara, Rocío Arco, Laura Orio, Juan Suárez, Beat Lutz, Raquel Gómez de Heras, Laura Bindila, Fernando Rodríguez de Fonseca

**Affiliations:** 1 Departamento de Psicobiología. Facultad de Psicología, Universidad Complutense de Madrid. Campus de Somosaguas s/n, Pozuelo de Alarcón, Madrid, Spain; 2 IBIMA, Unidad de Gestión Clínica de Salud Mental, Hospital Regional Universitario de Málaga, Universidad de Málaga, Málaga, Spain; 3 Institute of Physiological Chemistry, University Medical Center of the Johannes Gutenberg University of Mainz, Mainz, Germany; 4 Departamento de Biología Celular, Genética y Fisiología. IBIMA. Facultad de Ciencias, Universidad de Malaga. Campus de Teatinos s/n, Malaga, Spain; INIA, SPAIN

## Abstract

Undernutrition during pregnancy has been associated to increased vulnerability to develop metabolic and behavior alterations later in life. The endocannabinoid system might play an important role in these processes. Therefore, we investigated the effects of a moderate maternal calorie-restricted diet on the levels of the endocannabinoid 2-arachidonoyl glycerol (2-AG), arachidonic acid (AA) and the N-acylethanolamines (NAEs) anandamide (AEA), oleoylethanolamide (OEA) and palmitoylethanolamide (PEA) in the brain of newborn rat offspring. We focused on brain structures involved in metabolism, feeding behavior, as well as emotional and cognitive responses. Female Wistar rats were assigned during the entire pregnancy to either control diet (C) or restriction diet (R), consisting of a 20% calorie-restricted diet. Weight gain and caloric intake of rat dams were monitored and birth outcomes were assessed. 2-AG, AA and NAE levels were measured in hypothalamus, hippocampus and olfactory bulb of the offspring. R dams displayed lower gain weight from the middle pregnancy and consumed less calories during the entire pregnancy. Offspring from R dams were underweight at birth, but litter size was unaffected. In hypothalamus, R male offspring displayed decreased levels of AA and OEA, with no change in the levels of the endocannabinoids 2-AG and AEA. R female exhibited decreased 2-AG and PEA levels. The opposite was found in the hippocampus, where R male displayed increased 2-AG and AA levels, and R female exhibited elevated levels of AEA, AA and PEA. In the olfactory bulb, only R female presented decreased levels of AEA, AA and PEA. Therefore, a moderate diet restriction during the entire pregnancy alters differentially the endocannabinoids and/or endocannabinoid-related lipids in hypothalamus and hippocampus of the underweight offspring, similarly in both sexes, whereas sex-specific alterations occur in the olfactory bulb. Consequently, endocannabinoid and endocannabinoid-related lipid signaling alterations might be involved in the long-term and sexual dimorphism effects commonly observed after undernutrition and low birth weight.

## Introduction

Decades ago, Barker and colleagues demonstrated a strong and paradoxical correlation between low birth weight and the development of metabolic syndrome in adulthood [1]. Simultaneously, Dutch Famine cohort studies showed the long-lasting and deleterious effects of undernutrition during early development [2]. These investigations led to propose the DOHaD (Developmental origin of Health and Disease) hypothesis, stating that early life insults could lead to increased vulnerability to develop diseases later in life [1] through a process known as programming [3]. Extensive investigations in this area have focused on the effects of undernutrition in the fetal period. Particularly, it has been shown that poor nutritional environment in pregnancy is commonly associated to low birth weight, and to the development of metabolic diseases, such as obesity and metabolic syndrome [1], whose prevalence is reaching epidemic proportions worldwide [4].

Currently, although overnutrition is much more common in developed countries, the consequences of undernutrition in critical windows of development represent still a burden. For instance, for women in rich societies, the pressure of being fit and thin may lead to gain less weight than recommended, increasing the risk to deliver a baby small for his gestational age [5, 6]. Similarly, women with a past of eating disorders are at high risk for suffering preterm birth and intrauterine growth restriction fetuses [7]. Despite the risk of metabolic diseases, underweight at birth has been associated to behavioral abnormalities, including alterations in cognitive performance, inadequate emotional responses or modifications in feeding behavior [8–10]. Therefore, this evidence emphasizes the importance to approach the burden of fetal undernutrition from different perspectives.

The effects of malnutrition during critical windows of human development by using animal models mostly focused on investigating metabolic and/or behavioral alterations [11–14]. Similarly to human studies, investigations using different animal species, but predominantly rodents, have demonstrated that the phenotype exhibited by offspring following undernutrition in utero may depend on the sex [11, 15–17] but also on the developmental stage where undernutrition occurs [2, 18–20]. Furthermore and importantly, the research using animal models has highlighted the underlying mechanisms leading to inadequate programming, showing alterations in brain structures involved in metabolism, learning and emotional processes after exposure to fetal undernutrition. For instance, the impairment of hypothalamic circuitry development, intimately connected to modifications in leptin signaling, has been described in animal models of intrauterine growth restriction [21, 22]. Moreover, dysregulation in hippocampal circuitries associated to BDNF (brain-derived neurotrophic factor) alterations in specific developmental stages has also been reported in offspring, either after exposure to a maternal calorie-restricted diet [23] or low dietary intake of n-3 polyunsaturated fatty acids (PUFAs) during pregnancy and lactation [24].

Closely related to leptin signaling and BDNF [25, 26], the endocannabinod system (ECS), a lipid signaling system, has emerged as a putative modulator of the biological mechanisms involved in developmental programming [27]. Indeed, the ECS has been demonstrated to be crucial for regulating energy balance and food intake via central and peripheral mechanisms [28], as well as for the control of emotional responses and learning [29]. Consequently, ECS dysregulation has been associated to the development of obesity, metabolic syndrome and neuropsychiatric disorders [30, 31], which are diseases that might occur as a result of inadequate early life programming [1, 2, 8–13, 15–17, 20], as mentioned above. In addition to the endocannabinoids, non-cannabinoid acylethanolamines (OEA, PEA) that shares biosynthetic and degradation enzymatic pathways with anandamide, also contribute to the control of appetite, weight gain and lipid metabolism [28, 31, 32]

The main endocannabinoids anandamide (AEA) and 2-arachidonoylglycerol (2-AG), are synthesized from phospholipids containing arachidonic acid (AA), which is a linoleic acid derivative [32] and belongs to the n-6 polyunsaturated fatty acid (PUFA) family. Noteworthy, several investigations have revealed the importance of PUFAs in brain development. For instance, a negative correlation between n-3 PUFA intake and increased vulnerability to neuropsychiatric disorders has been shown [33–36]. The role of n-6 PUFAs, precursors of arachidonic acid and ultimately of endocannabinoids, has been pointed out as critical in these processes as well [33, 37]. Specifically, endocannabinoid signaling plays a crucial role in important processes involved in brain maturation, including the establishment of adequate neural connections and synaptogenesis [33, 38]. Moreover, prenatal administration either of agonists, such as THC (Δ^9^-tetrahydrocannabinol), or antagonists of cannabinoid receptors, has been associated to disruption of neuronal activity, defective establishment of cortical connectivity and behavioral abnormalities [39–43].

Although less investigated, altered nutritional conditions during early life might also have an impact on endocannabinoid signaling, leading to disturbances in brain functions and/or behaviors. Thus, prenatal and postnatal exposure to restricted omega-3 diet has been associated to impaired endocannabinoid-mediated neuronal functions in the adult brain, together with behavioral abnormalities [36]. Moreover, exposure to a maternal diet rich in n-3 or n-6 fatty acids modifies arachidonic acid and/or endocannabinoid levels in neonatal hypothalamus and hippocampus, resulting in alterations in the hypothalamus- pituitary-adrenal axis functions [44]. Therefore, this piece of evidence suggests that an inadequate endocannabinoid signaling resulting from exposure to an unbalanced maternal diet, might disrupt the establishment of functional circuitries involved in metabolism, learning and emotional control, leading to metabolic and neurobehavioral abnormalities later in life [27].

To date, only a few studies have addressed the relation between a global undernutrition in early life and endocannabinoid signaling. For instance, a pioneer study demonstrated modifications in the levels of endocannabinoids at weaning after maternal exposure to a calorie-restricted diet during pregnancy and/or lactation [45]. However, the impact of nutrient deficiency in earlier stages has been poorly investigated. Addressing this question could be especially pertinent considering that endocannabinoid levels fluctuate strongly during early development [46], which suggests a potentially critical contribution of endocannabinoid signaling in the earliest neural development processes. Accordingly, we have recently showed that exposure to a hypocaloric maternal diet implemented before and during gestation has an impact on the brain endocannabinoids and endocannabinoid-related lipids, leading to long-lasting consequences in offspring [47].

Taking into account that the timing of caloric restriction could be critical on the effects exhibited by offspring [2, 18–20] and that these effects might be sex-dependent [11, 15–17], this study aims at investigating the impact of a moderate caloric restriction applied during the entire pregnancy on male and female newborn rats. Particularly, we measured at birth the endocannabinoid, arachidonic acid and N-acylethanolamines (NAEs) content in brain structures involved in the modulation of metabolism, feeding behavior, learning and emotional responses, such as hypothalamus, hippocampus and olfactory bulb [28, 29, 48]. We hypothesize that endocannabinoid signaling could be impaired in the offspring after exposure to maternal undernutrition during the complete pregnancy in a sex specific-manner.

## Material and methods

This study was approved by the Animal Ethics Committee of the Complutense University of Madrid and was conducted in compliance with the European Directive 2010/63/EU on the protection of animals used for scientific purposes and according to the Spanish regulations (RD 53/2013 and 178/2004).

### Animals, diets and experimental design

Adult female Wistar rats (6 months old) (Harlan, Barcelona, Spain) were allowed to acclimate for a minimum of four weeks before the beginning of the experiments. Rats were handled and housed in groups under a 12 hours light-dark cycle with temperature of 22±1°C. After the acclimation period, animals weighed 304±4 g and estrous cycle was evaluated daily. In the morning of proestrous, females were allowed to mate with a male of the same strain. Each male rat was mated with females from both groups (described below). The mating phase lasted 24 hours and occurred in the female cage. In the following morning the presence of vaginal plug or spermatozoa in vaginal smear confirmed successful mating, and this was defined as gestational day 0. Then, females rats were individually housed and randomly assigned to control (n = 4) or caloric restriction diet (n = 7) groups. At this stage, no statistical significant difference in body weight between groups was found.

Control rats (n = 4) were given free access to standard chow (Standard chow SAFE A04, Panlab, Barcelona, Spain). The standard chow provided 16.1% protein, 60% carbohydrate, 3.1% fat, 4% fiber, 0.0025% sodium and 2.9 kcal/g as energy content. In contrast, calorie-restricted dams (n = 7) were given a daily amount of food corresponding to 80% of the calories provided to control rats in the same gestational day, according to body weight (20% of caloric restriction). Water was provided ad libitum in both animal groups.

The day the dams were found with their respective litter was defined as postnatal 0 (PN0). Within 14 hours after birth, pups were weighed, sexed and sacrificed by quick decapitation. Brains were collected and brain regions were dissected for endocannabinoids measurement. None of the animals utilized in the present study showed signs of illness or died prior to the experimental endpoint.

### Endocannabinoids and endocannabinoid related-lipids measurement

At PN0, male offspring chosen to be sacrificed were decapitated during the second/third hour of the dark phase and brains were quickly removed and frozen at −80°C until brain region dissection. To avoid the possibility of variable outcomes among litters, brains from at least three litters per group were used to carry out endocannabinoids and endocannabinoid related lipid measurement (control male pups, n = 14-14-12 and male pups from calorie-restricted group, n = 14-18-14, for hypothalamus, hippocampus and olfactory bulb respectively; control female pups, n = 10-9-9 and female pups from calorie-restricted group, n = 10-10-10, for hypothalamus, hippocampus and olfactory bulb respectively). For the isolation of the selected brain regions, brains were thawed in cold Tris-HCl buffer (50 mM, pH = 7.4) and the entire hypothalamus, right hippocampus and right olfactory bulb was quickly dissected and immediately frozen at −80°C until lipid extraction. The overall dissection procedure was carried out in less than 7 minutes for all animals to allow reliable comparative assessment of endocannabinoid levels.

For lipid extraction, pre-cooled steel balls of 5 mm were added to pre-cooled tubes containing the tissue. A solution of deuterated endocannabinoids (AEA-d4, 2-AG-d5, AA-d8, MAEA, OEA-d2, PEA-d4 and 1-AG-d5, Cayman Chemicals, Ann Arbor, MI, USA) in acetonitrile was added to the tissue along with 300 μL of ice-cold 0.1 M formic acid and 300 μl of ethylacetate/hexane (9:1, v/v). Then, the samples were homogenized with a TissueLyser II (Qiagen, Hilden, Germany) for 60 s at 30 Hz. Subsequently, the samples were centrifuged for 10 min at 5,000 g and 4°C and frozen at -20°C for 20 min. The organic phase was removed and evaporated under a gentle stream of nitrogen at 37°C. The aqueous phase was further used for protein content determination. The lipid extract was resolubilized in 50-μL acetonitrile/water (1:1, v/v) and quantitative analysis of the endocannabinoid levels was carried out by liquid chromatography-multiple reaction monitoring (LC-MRM). The concentrations of internal standards, as well as the calibration curves, were set and tailored using test hypothalamus, hippocampal and olfactory bulb tissues. The LC/MRM conditions for quantitative analysis of the endocannabinoids were set as previously described [49]. For protein quantification, the BCA method (bicinchoninic acid assay) was used and measurements were performed by using a FLUOstar Galaxy (BMG Labtechnologies).The endocannabinoid levels determined by LC/MRM were then normalized to the corresponding protein content of the tissues as previously described [49–51].

### Statistical analysis

Caloric intake and body weight gain over time of rat dams were analyzed by two-way repeated measures analysis of variance (ANOVA), with time and pregnancy diet as factors. Multiple comparisons were assessed by Bonferroni post hoc test. Further analysis were performed by using the Student’s *t*-test, when data passed the normality requirements (D’Agostini Pearson test), or Mann-Whitney’s U test. A *p*-value below 0.05 was considered statistically significant.

## Results

### Impact of a moderate caloric restriction during gestation on rat dams

#### Effect of a moderate gestational restriction diet on maternal weight gain

**Fig 1:**
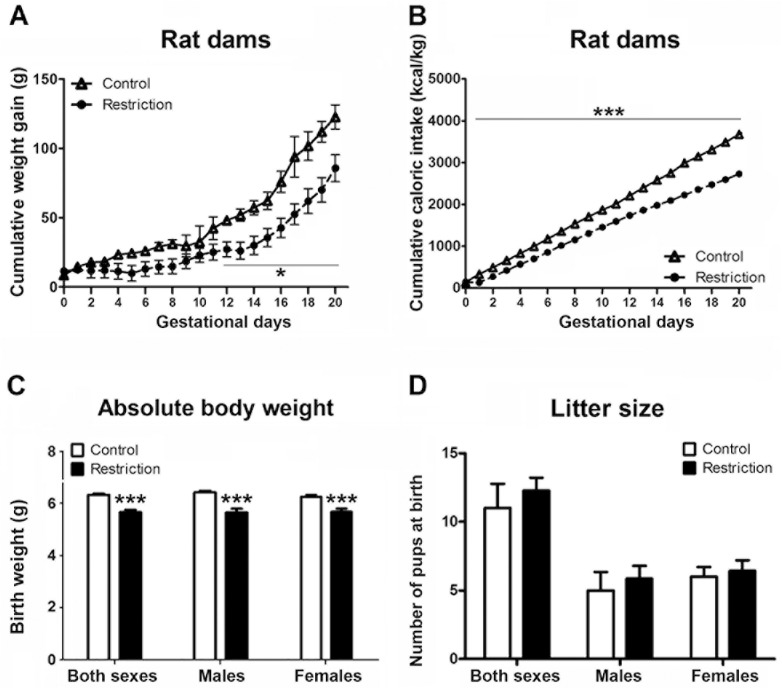
Effect of gestational calorie-restriction on rat dams and birth outcomes. Experiments started the following day of mating. Calorie-restricted rat dams (n = 7) received 80% of control dams (n = 4) food intake according to body weight, which was measured daily (restriction of 20%). Calorie-restricted diet lasted until birth. Figures A and B describe the cumulative weight gain (g) and cumulative caloric intake (Kcal/Kg), respectively, of control (open triangles) and calorie-restricted (solid circles) dams during pregnancy. At PN0 (birth day), litter size was evaluated and pups were sexed and weighed. Figures C and D describe the absolute body weight (g) and litter size, respectively, of offspring from control dams (n = 30) and offspring from calorie-restricted dams (n = 47) at birth (open and solid bars, respectively). Values are expressed as mean +/- SEM. Data were analyzed with repeated measures ANOVA followed by Bonferroni multiple comparisons (A, B), and Student´*t* test (C, D): *p<0.05, ***p<0.001.

Repeated measures ANOVA showed decreased cumulated weight gain in calorie-restricted dams as compared to controls during the entire pregnancy (F(_1,9_) = 5.7, p<0.05). Specifically, Bonferroni multiple comparisons showed that statistically significant differences between groups started at gestational day 12 (F(_1,9_) = 7.78, p<0.05) and lasted up to day 20 (F(_1,9_) = 6.40, p< 0.05) (**Fig 1A** and **S1 Data**). Moreover, at PN0, calorie-restricted mothers weighed significantly less than controls (mean weight and SEMs of controls vs calorie-restriction: 342.1 9.38 vs 303.2 9.82, Mann-Whitney’s U test, U(4,7) = 3, p<0.05) (data not shown).

#### Effect of a moderate gestational restriction diet on maternal caloric intake

According to the experimental design carried out, the cumulative caloric intake of calorie-restricted dams was decreased (repeated measures ANOVA, F_(1,9)_ = 184.51, p<0.001). Statistically significant differences between groups started at gestational day 1 (F_(1,9)_ = 53.08, p<0.001) and lasted up to the end of measurements (day 20) (F_(1,9)_ = 169.53, p<0.001) (**Fig 1B**).

Taken together these data indicate that calorie-restricted diet has an impact on weight gain and absolute body weight during pregnancy. Moreover, taking into account the experimental design adopted in the present study, calorie-restricted dams consumed less calories as compared to controls.

### Effect of a moderate maternal caloric restriction on birth outcomes

Pups from control dams and calorie-restricted mothers were born between gestational day 21–22. At birth, offspring from gestational calorie-restricted dams weighed significantly less than controls: both sexes taken together (t = 6.199, p<0.001); male (t = 4.768, p<0.001); female (t = 3.997, p<0.001) (**Fig 1C**). In contrast, no significant differences in litter size were found either in both sexes analyzed together or in each sex analyzed separately (**Fig 1D**). Thus, gestational calorie-restriction leads to underweight at birth without modifying the litter size.

### Impact of a moderate maternal caloric restriction on endocannabinoid and endocannabinoid-related lipid levels in specific brain regions of male and female offspring at birth

#### Hypothalamic endocannabinoid and endocannabinoid-related lipid levels in male and female offspring at birth

**Fig 2:**
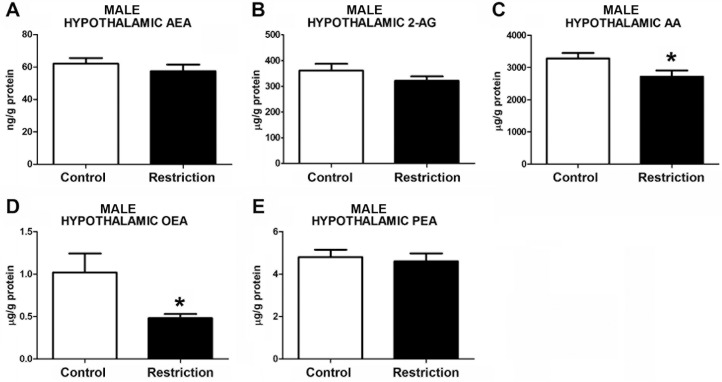
Endocannabinoid and endocannabinoid-related lipid levels in the hypothalamus of male offspring at birth. Anandamide (AEA), 2-arachidonoylglycerol (2-AG), arachidonic acid (AA), oleoylethanolamide (OEA) and palmitoylethanolamide (PEA) levels in the hypothalamus of male offspring (A-E) from control dams (n = 14) and calorie-restricted dams (n = 14) at birth (open bars and solid bars, respectively). Values are expressed as mean +/- SEM. Data were analyzed by Student´s *t*-test (A, B) or Mann Whitney´s U test (C, D, E): *p<0.05.

**Fig 3:**
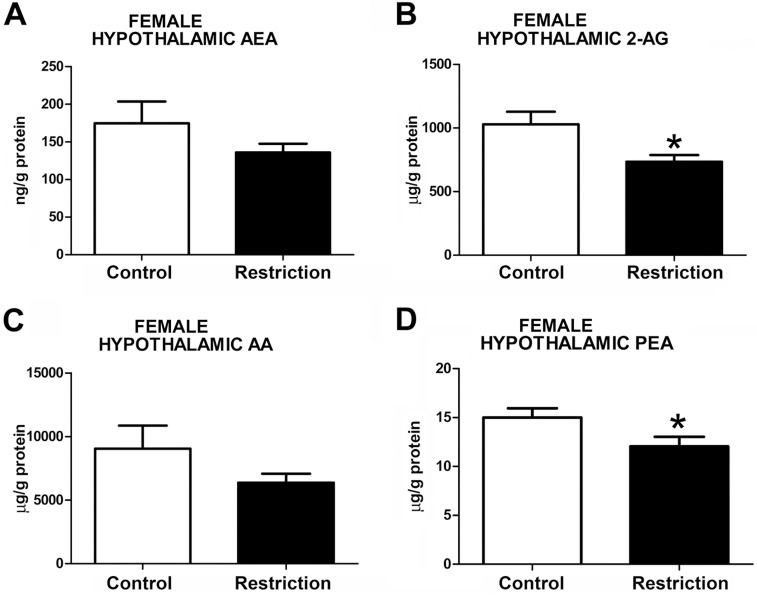
Endocannabinoid and endocannabinoid-related lipid levels in the hypothalamus of female offspring at birth. Anandamide (AEA), 2-arachidonoylglycerol (2-AG), arachidonic acid (AA) and palmitoylethanolamide (PEA) levels in the hypothalamus of female offspring (A-D) from control dams (n = 10) and calorie-restricted dams (n = 10) at birth (open bars and solid bars, respectively). Values are expressed as mean +/- SEM. Data were analyzed by Student´s *t*-test (B, D) or Mann Whitney´s U test (A, C): *p<0.05.

Statistically significant differences between perinatal groups were found in endocannabinoids and/or endocannabinoid-related lipids at birth in male and female offspring. Specifically, male pups from gestational calorie-restricted dams displayed significant lowers levels of AA as compared to controls (U = 45.00, p<0.05) (**Fig 2C** and **S2 Data**), but similar levels of AEA (t = 0.8515, p>0.05) and 2-AG (t = 1.275, p>0.05) (**Fig 2A and 2B**, respectively). Regarding NAEs levels, offspring from calorie-restricted dams presented lower concentrations of oleoylethanolamide (OEA) (U = 46.00, p<0.05) (**Fig 2D**), but no significant differences in palmitoylethanolamide (PEA) levels (U = 79, p>0.05) (**Fig 2E**). Female pups exhibited decreased level of 2-AG (t = 2.649, p<0.05) (**Fig 3B**) but no differences either in AEA or AA (U = 42, p>0.05 and U = 36, respectively) were found (**Fig 3A and 3C**). Females also presented a reduction of PEA levels (t = 2.197, p<0.05) (**Fig 3D**). The OEA values in the hypothalamus could not be reliably quantified in female offspring (data not shown).

Taken together, these data show that a moderate caloric restriction diet during pregnancy decreases hypothalamic content of the endocannabinoid and/or the endocannabinoid-related lipids in the offspring with sex-dependent differences.

#### Hippocampal endocannabinoid and endocannabinoid related-lipid levels in male and female offspring at birth

**Fig 4:**
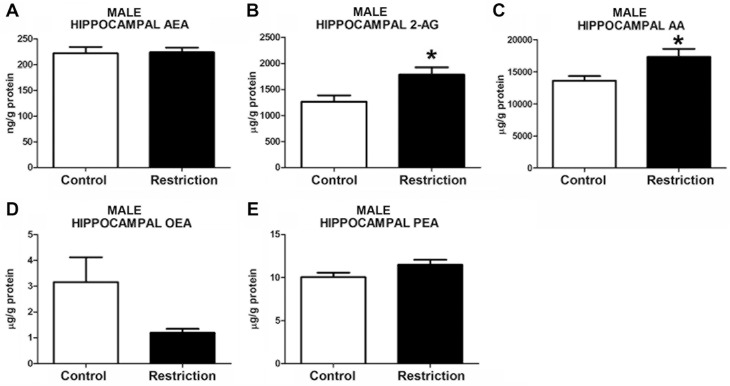
Endocannabinoid and endocannabinoid-related lipid levels in the hippocampus of male offspring at birth. Anandamide (AEA), 2-arachidonoylglycerol (2-AG), arachidonic acid (AA), oleoylethanolamide (OEA) and palmitoylethanolamide (PEA) levels in the hippocampus of male offspring (A-E) from control dams (n = 14) and calorie-restricted dams (n = 18) at birth (open bars and solid bars, respectively). Values are expressed as mean +/- SEM. Data were analyzed by Student´s *t*-test (A, B, E) or Mann Whitney´s U test (C, D): *p<0.05

**Fig 5:**
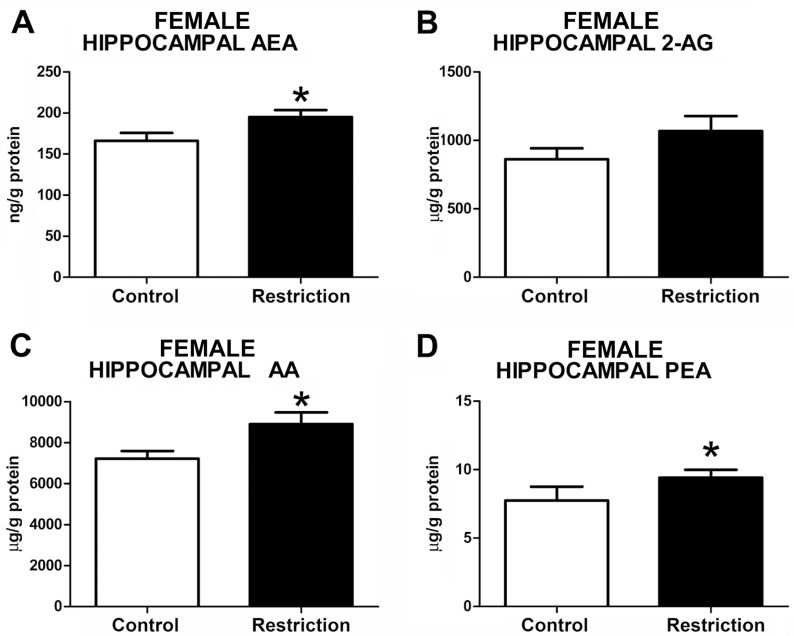
Endocannabinoid and endocannabinoid-related lipid levels in the hippocampus of female offspring at birth. Anandamide (AEA), 2-arachidonoylglycerol (2-AG), arachidonic acid (AA), and palmitoylethanolamide (PEA) levels in the hippocampus of female offspring (A-D) from control dams (n = 9) and calorie-restricted dams (n = 10) at birth (open bars and solid bars, respectively). Values are expressed as mean +/- SEM. Data were analyzed by Student´s *t*-test (A, B,C) or Mann Whitney´s U test (D): *p<0.05.

Measurements of hippocampal endocannabinoid and endocannabinoid-related lipids showed statistical differences between perinatal groups in both sexes. Specifically, the male offspring from calorie-restricted dams displayed increased levels of 2-AG (t = 2.721, p<0.05) and AA (U = 65.00, p<0.05) (**Fig 4B and 4C**, respectively, and **S3 Data**). A strong tendency to increased levels of PEA in calorie-restricted male offspring was also detected (t = 1.775, p = 0.08) (**Fig 4E**). However, no differences between groups either in AEA (t = 0.1325, p>0.05) or OEA (U = 93, p>0.05) levels were found (**Fig 4A and 4D**, respectively). In contrast, calorie-restricted female pups showed increased hippocampal AEA (t = 2.264, p<0.05) (**Fig 5A**) and, similarly to male offspring, enhanced levels of AA (t = 2.401, p<0.05) (**Fig 5C**), although 2-AG levels were unchanged (t = 1.489, p>0.05) (**Fig 5B**). Moreover, female offspring from diet-restricted dams presented higher hippocampal levels of PEA (U = 18, p<0.05) than control female pups (**Fig 5D**). The OEA values in the hippocampus could not be reliably quantified in female offspring (data not shown).

Taken together, these data indicate that a moderate caloric restriction during pregnancy increases the hippocampal endocannabinoids and/or endocannabinoid-related lipids in the offspring with sex-dependent differences.

#### Endocannabinoid and endocannabinoid-related lipid levels in the olfactory bulb of male and female offspring at birth

**Fig 6:**
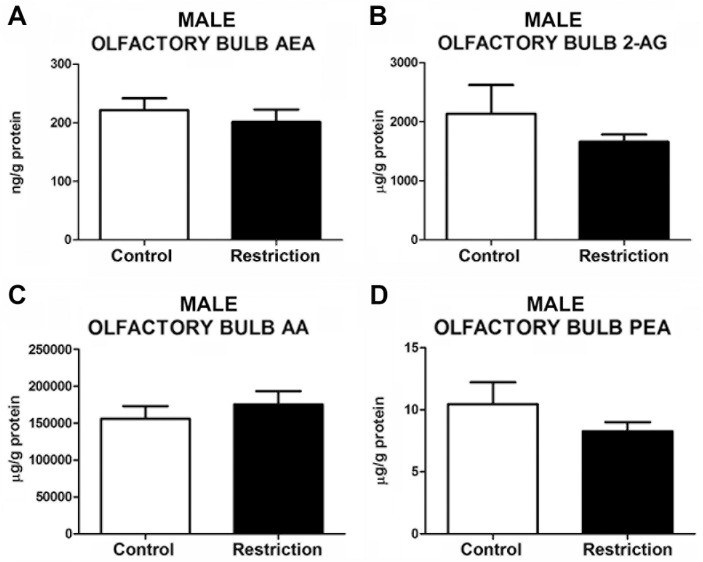
Endocannabinoid and endocannabinoid-related lipid levels in the olfactory bulb of male offspring at birth. Anandamide (AEA), 2-arachidonoylglycerol (2-AG), arachidonic acid (AA) and palmitoylethanolamide (PEA) levels in the olfactory bulb of male offspring (A-D) from control dams (n = 12) and calorie-restricted dams (n = 14) at birth (open bars and solid bars, respectively). Values are expressed as mean +/- SEM. Data were analyzed by Student´s *t*-test (A) or Mann Whitney´s U test (B, C, D): *p<0.05.

**Fig 7:**
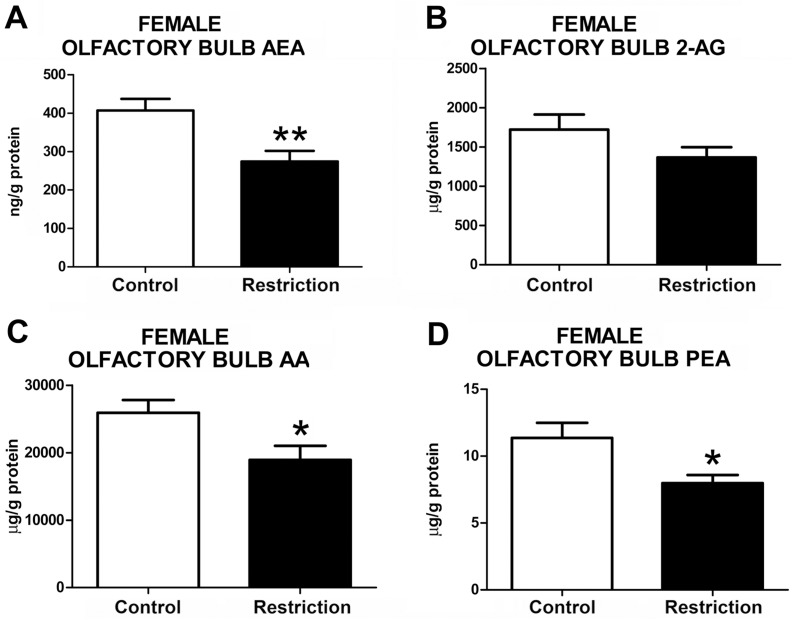
Endocannabinoid and endocannabinoid-related lipid levels in the olfactory bulb of female offspring at birth. Anandamide (AEA), 2-arachidonoylglycerol (2-AG), arachidonic acid (AA) and palmitoylethanolamide (PEA) levels in the olfactory bulb of female offspring (A-D) from control dams (n = 9) and calorie-restricted dams (n = 10) at birth (open bars and solid bars, respectively).Values are expressed as mean +/- SEM. Data were analyzed by Student´s *t*-test: *p<0.05, **p<0.01.

The statistical analysis did not reveal any alteration in endocannabinoids, such as AEA (t = 0.68, p>0.05) and 2-AG (U = 75, p>0.05), AA (U = 68, p>0.05) and PEA (U = 68, p>0.05) levels in the olfactory bulb of male offspring from calorie-restricted dams as compared to controls (**Fig 6**). In contrast, significant alterations in the endocannabinoid and endocannabinoid-related lipids were detected in female restricted offspring (**Fig 7** and **S4 Data**). Specifically, calorie-restricted females exhibited decreased levels of AEA (t = 3.279, p<0.01) (**Fig 7A**), AA (t = 2.471, p<0.05) (**Fig 7C**) and PEA (t = 2.639, p<0.05) (**Fig 7D**) in this brain structure. No differences were found between groups in the levels of 2-AG (t = 1.550, p<0.05) (**Fig 7B**). The OEA values in the olfactory bulb could not be reliably quantified in both male and female offspring (data not shown).

Taken together, these data indicate that a moderate caloric restriction diet during pregnancy modifies the levels of endocannabinoids and/or endocannabinoid-related lipids in the hypothalamus, hippocampus and/or olfactory bulb of offspring. Specifically, male and female offspring from calorie-restricted dams that were underweight at birth displayed decreased endocannabinoids and endocannabinoids-related lipids in the hypothalamus, whereas the opposite was found in the hippocampus. The female offspring also showed the same tendency as hypothalamus to reduced endocannabinoid and endocannabinoid-related lipids in the olfactory bulb. Importantly, alterations in each endocannabinoid and/or related lipid occurred differently according to the sex of the offspring.

## Discussion

The main finding of the present study is that newborn rats exposed to a moderate caloric restriction during the entire pregnancy displayed alterations in endocannabinoids and/or endocannabinoid-related lipids, in brain structures involved in the regulation of metabolism and emotional and cognitive responses. Specifically, male and female offspring from diet-restricted dams exhibited decreased levels of the main endocannabinoids, their precursor and/or NAEs in the hypothalamus and, conversely, they showed enhanced content of these lipids in the hippocampus. This similar profile between males and females from calorie-restricted dams was not evident in the olfactory bulb, where the calorie-restricted female offspring presented decreased levels of AEA, their precursor (AA) and PEA. Moreover, these modifications were accompanied of underweight at birth, a common result when diet restriction is applied for the entire pregnancy or in the last phases of pregnancy [12, 13]. Interestingly, this finding has been widely associated to metabolic and behavioral abnormalities later in life [12–14].

The majority of animal research conducted to evaluate the effects of undernutrition in critical developmental windows has focused on investigating the deleterious effects of a severe gestational diet restriction in the offspring [12, 13]. In our study, we have adopted a moderate maternal calorie-restriction, that may have simulated better the reduction in food intake documented in some human studies, commonly associated to decreased weight gain during pregnancy [5, 6, 52], and could have prevented unnecessary effects in the animals. In rodents, this type of maternal restriction has been demonstrated previously to be enough to induce long-lasting alterations in offspring [11, 14, 53, 54]. Moreover, we have recently showed that the preconceptional and gestational exposure to a moderate calorie-restricted diet increases the risk of developing features of metabolic syndrome as well as behavioral abnormalities in the offspring [47, 55]. Importantly, in the present study, we have demonstrated for the first time that a moderate maternal caloric restriction applied during the entire pregnancy alters brain endocannabinoid and/or endocannabinoid-related lipid levels at birth in male and female offspring and reduces the weight at birth.

The modifications in the supply of nutrients to the fetus may have altered the intrauterine growth leading to inadequate size at birth in our study. Regarding the most important nutrients during intrauterine life, apart from glucose and aminoacids, the fatty acids and, particularly, the long-chain (LC) PUFAs, such as the arachidonic acid (AA) and docosahexaenoic acid (DHA) has been revealed as critical elements for a correct growth and neurodevelopment [37, 56]. The concentration of LC-PUFAs and their precursor depends on diet, fatty acid storage in the adipose tissue and endogenous synthesis, which requires adequate functionality of the enzymes involved in desaturation and elongation of essential fatty acids [57]. Therefore, to ensure an adequate fatty acid supply, the fetuses and new born animals depend on the mother nutritional status and the placenta functionality to obtain both essential fatty acids (EFAs) and long-chain fatty acids (LC-PUFAs). This is due to the limited capacity of the fetus to transform EFAs and the importance of depositing the PUFAs in key tissues, including the fetal brain, during intrauterine growth [57, 58]. Regarding the endocannabinoids, little is known about the fetal-maternal relationship in endocannabinoid content. It has been proposed that, although n-6 PUFA derivatives may be synthetized by the fetus in the tissues, the placenta may also transfer maternal endocannabinoids to the fetus by contributing to maintenance of the endocannabinoid basal tone [27]. Consequently, a maternal caloric restriction in our study may have had an impact on the levels of PUFAs and their derivatives, including the endocannabinoids, in the fetus, and leading to alterations in endocannabinoid and related lipid levels in different brain structures.

Indeed, we found decreased levels of endocannabinoids and/or endocannabinoid-related lipids in the hypothalamus of male and female offspring. Specifically, we found decreased AA levels in the hypothalamus of male offspring, without any change in the concentrations of AA-derived endocannabinoids (i.e., AEA and 2-AG). In contrast, the female offspring exhibited decreased levels of 2-AG in this brain region despite the unchanged concentrations of its precursor (AA). Apart from the reduced concentration of the LC-PUFAs after a caloric restriction, including the reduction of the AA, precursor of endocannabinoids, an alternative explanation to these findings may involve a sex-specific alteration in the activity and/or levels of the endocannabinoid metabolic enzymes. Therefore, in an attempt to maintain endocannabinoid and/or AA homeostasis, an increased synthesis of 2-AG and AEA could have lately determined the reduction of AA in male offspring. Conversely, an increased degradation of 2-AG may have determined its reduction and maintained the AA content in females unchanged. Further analysis of the expression and activity of enzymes responsible for biosynthesis and degradation of endocannabinoids has to be addressed to confirm this hypothesis. Another possibility is that an increased endocannabinoid transfer from mother, through placenta, might have also contributed to maintain an endocannabinoid tone in the hypothalamus of male offspring [27], despite the decreased AA availability and the depletion in maternal tissues due to undernutrition. This idea is supported by previous studies showing differences in male and females placentas after inadequate maternal diets [59, 60].

Our finding in male hypothalamus contrasts with a previous report [45], describing decreased hypothalamic endocannabinoid levels in male offspring coming from dams exposed to a 50% calorie-restricted diet during late pregnancy. However, in this interesting study and differently to ours, the endocannabinoid measurement was performed at weaning, and not at birth. Considering that the pups are independent from the maternal fatty acid stores at this developmental stage, the levels of endocannabinoids here may easily have reflected the metabolic status of the pups and, thus, their own fatty acid stores. Specifically, in this previous study the pups at weaning exhibited decreased body weight that was accompanied by a reduction of hypothalamic AEA. Although the body weight at birth was not reported in this study, similar types of restriction have been associated to decreased body weight at birth, which suggests that these animals did not exhibited a rapid catch-up growth. This phenomenon has been described after caloric restriction in pregnancy and/or small fetuses associated to hyperphagia [12, 13] and is known to induce a severely dysfunctional metabolic phenotype in the offspring later life [13, 61]. Therefore, the decreased hypothalamic anandamide described in the study of Matias et al. (2003) might have affected the appetite [62, 63] in these animals, leading to a lean phenotype at weaning and favoring complete recovery at adulthood. Although we did not evaluate the long-term effects on the offspring, the opposite might have occurred in our study. Particularly, we found decreased levels of OEA in the hypothalamus of male offspring from calorie-restricted mothers. Considering that this NAE is involved in the modulation of satiety [64], our data suggest a possible increased appetite in these animals, similar to previously reported in offspring exposed to undernutrition in pregnancy and undergoing to rapid catch-up growth during lactation and infant period and impaired metabolic phenotype at adulthood [13, 61].

Moreover, in female hypothalamus we found decreased levels of PEA, a NAE with anti-inflammatory and antiobesity properties [65, 66], suggesting the possibility of inflammatory status in female offspring, which has been associated to the development of metabolic and behavioral abnormalities [67–69].

The reduction of the levels of OEA and PEA in male and female offspring may be a consequence of a limited amount of the precursors required for their synthesis, particularly the oleic acid and/or palmitic acid, as previously proposed in adult animals [70, 71] and recently demonstrated in humans [72]. Additionally, the impairment of the activity and/or levels of the enzymes implicated in the synthesis and degradation of NAEs might also explain these results. Further research is needed to address these possibilities.

Concerning the possible interpretation to our findings in hypothalamus, it is important to note that previous studies have showed that the reduction in both AA and endocannabinoid levels in this brain structure at birth is associated to the development of metabolic disturbances at adulthood [47, 51]. Although alterations in endocannabinoids were not observed in male offspring in this brain region, the decreased levels of AA and their association with decreased birth at weight found in the present study might have promoted adverse consequences in the development of hypothalamus. This supposition takes into account that AA is a LC-PUFA involved in growth and brain development and is the precursor of the eicosanoids, which contribute to regulate cell proliferation, growth, immunity and inflammation [37].

We also evaluated the endocannabinoid levels in the hippocampus, a brain structure involved in modulating emotional responses and where the endocannabinoid system plays an important role in memory formation and neurogenesis associated to metabolism-dependent mechanisms [29, 73]. Intriguingly, we found increased hippocampal levels of AA and 2-AG at birth in male offspring from calorie-restricted dams, which is opposite to the findings in the hypothalamus. In the female hippocampus we also found increased concentration of AEA, AA and PEA levels. The increased endocannabinoid and AA levels found in our animals might reflect a fetal adaptation against the reduced availability of nutrients to preserve hippocampal development. This idea is supported by previous works showing fluctuating BDNF levels, a protein related to endocannabinoids [26], in different stages of the brain development of offspring from severely calorie-restricted dams [23]. Interestingly, a peak in 2-AG levels at PN1 in the whole brain has been described together with increased expression of the cannabinoid receptor type 1 (CB_1_), suggesting the importance of endocannabinoids (i.e., 2-AG) and endocannabinoid synthesis precursors (i.e., AA) for an adequate brain development [46]. Considering this evidence, the increased 2-AG and AA levels at PN0 in male offspring may reflect a premature peak to prevent the deleterious effects on hippocampus development. In female offspring we found increased AEA and PEA levels. It is interesting to note that the enzyme fatty acid amide hydrolase (FAAH) degrades both AEA and PEA [74], which suggests that an eventual alteration in this metabolic enzyme may be implicated. Moreover, the increased levels of AA, 2-AG and AEA in male and female offspring suggest that implementing a moderate caloric restriction in rat previously well-nourished might have a modest impact on the fatty acid storage at the beginning of pregnancy, favoring the deposition of AA in hippocampus. In particular, it is interesting to note that these findings contrasts with the results recently described by our group in offspring from mothers exposed to the same caloric restriction during the preconceptional and gestational period, and with presumably decreased maternal stores. In this previous study, we observed decreased levels of AEA in hippocampus at birth in association to increased anxiety-related responses in adolescence [47], suggesting that the potential compensatory effect of increased endocannabinoids in the hippocampus is inverted in worse nutritional conditions. Further research is needed to confirm these possibilities.

Although the role of the endocannabinoid system in behavioral programming has not been well established yet, alterations in hippocampal endocannabinoid content are known to promote impaired emotional and cognitive responses. For instance, a decrease in the hippocampal 2-AG level has been correlated to anxiety-related responses [51, 75, 76], and the blockade of anandamide reuptake specifically in the hippocampus produces anxiolytic effects [77]. Furthermore, increased 2-AG in hippocampus was associated to mitigation of the cognitive alterations in severely undernourished mice supplemented with a diet rich in fish oil, an important source of n-3 PUFAs [78], although the opposite has been described in an animal model of schizophrenia [79]. In the context of nutritional programming, emotional responses and cognitive performance have been found to be affected after exposure to undernutrition during critical windows of development and/or in new born small for gestational age [14, 20, 80] and in a sex specific-manner [81, 82]. Despite this evidence, the increased levels of 2-AG, AEA and AA we found in the hippocampus are difficult to interpret, considering that CB_1_ receptor activation by endocannabinoids may mediate bimodal opposite responses depending on the differential distribution of CB_1_ in distinct neuronal populations [29].

Additionally, we measured endocannabinoid and NAE levels in the olfactory bulb of male offspring. The contribution of the endocannabinoid system has been revealed recently in this brain structure, where CB_1_ receptor stimulation increases odor perception and food intake in fasted animals [48]. In male offspring, we did not detect any modification in the endocannabinoid levels in the olfactory bulb from calorie-restricted dams, even though these animals displayed higher levels of OEA in the hypothalamus, which was probably associated to disrupted hunger and/or feeding behavior in these animals. However and interestingly, the female offspring displayed decreased levels of AEA, AA and PEA in the olfactory bulb, suggesting alterations in feeding behavior and an inflammatory status. The findings in female offspring are in agreement to a previous report showing that prenatal adverse conditions (such as prenatal stress) can affect odor preference in a sex specific-manner, leading to alterations in odor preference in female offspring [83] and suggesting that the females might have increased vulnerability in this brain structure after exposure to adverse perinatal conditions.

The changes in endocannabinoid and/or endocannabinoid-related lipid levels found in the hypothalamus and hippocampus of male and female offspring from calorie-restricted dams raise several questions. On the one hand, as modifications in these lipid regulators were found in developing brain structures in association with decreased weight at birth, we cannot discard the possibility that these alterations might have long-lasting consequences in the offspring, as it has been previously reported [12–14]. Indeed, alterations in endocannabinoid signaling in brain structures involved in the modulation of metabolism and emotional responses may lead to inadequate neuronal wiring or subtle alterations in neuronal connectivity and favor vulnerability to diseases later in life [27]. Moreover, previous studies have shown that alterations in endocannabinoid signaling during early development after treatment with specific agonists/antagonists of the CB_1_ cannabinoid receptors are associated to long-lasting neurochemical, endocrine and behavioral effects [39–43]. In support of this notion, we have recently reported changes in brain endocannabinoids and endocannabinoid-related lipids at birth after inadequate maternal diets in hypothalamus and hippocampus in association with metabolic and behavioral alterations [47, 51]. On the other hand, it is possible that some brain structures were protected from the effects of a moderate caloric restriction implemented only during pregnancy by the preferential uptake of the fetal tissues of specific LC-PUFAs, such as AA or n-3 PUFAs. It is interesting to note here that the n-3 PUFAs can affect the levels of endocannabinoids by decreasing their levels by competing for the metabolic enzymes [70, 84], or by increasing their levels depending on different circumstances [78, 85]. Additionally, the presence of sexual dimorphism mainly associated to the alterations found in olfactory bulb and the PEA levels in all the brain regions of females, suggest that the maternal calorie-restriction might have affected the male and female offspring through different mechanisms. Further investigations are needed to explore these possibilities.

## Conclusions

In summary, we have demonstrated that a moderate caloric restriction during the entire pregnancy results in underweight offspring with altered endocannabinoid, AA and/or NAE levels in the hypothalamus, hippocampus and/or olfactory bulb of male and female offspring at birth in a sex-specific manner. These data represent a first step towards understanding the possible contribution of the ECS in the nutritional programming, considering the available data on the long-lasting effects of undernutrition and underweight at birth. Understanding why dietary manipulations modify hypothalamic, hippocampal and olfactory bulb endocannabinoid and endocannabinoid-related lipid levels, and whether these changes lead to permanent dysfunctions in the ECS and/or impairment in circuitries involved in the regulation of metabolism and emotional behaviors in a sex-specific manner need to be elucidated. Therefore, further investigations are required to clarify the role of the ECS in nutritional programming.

## Supporting information

S1 Data Gestation weight gain in rat dams. (PZF)

S2 Data Endocannabinoid and endocannabinoid-related lipid levels in hypothalamus. (PZF)

S3 Data Endocannabinoid and endocannabinoid-related lipid levels in hippocampus. (PZF)

S4 Data Endocannabinoid and endocannabinoid-related lipid levels in olfactory bulb. (PZF)

## References

[1] Barker DJ. The origins of the developmental origins theory. J Intern Med. 2007;261(5):412–7. 10.1111/j.1365-2796.2007.01809.x 17444880

[2] Roseboom T, de Rooij S, Painter R. The Dutch famine and its long-term consequences for adult health. Early Hum Dev. 2006;82(8):485–91. 10.1016/j.earlhumdev.2006.07.001 16876341

[3] Lucas A. Programming by early nutrition in man. Ciba Found Symp. 1991;156:38,50; discussion 50–5. 1855415

[4] World Health Organization. Global status report of noncommunicable diseases 2014. Geneve: WHO Press, World Health Organization; 2014.

[5] DeVader SR, Neeley HL, Myles TD, Leet TL. Evaluation of gestational weight gain guidelines for women with normal prepregnancy body mass index. Obstet Gynecol. 2007;110(4):745–51. 10.1097/01.AOG.0000284451.37882.85 17906004

[6] Easter A, Bye A, Taborelli E, Corfield F, Schmidt U, Treasure J, et al Recognising the symptoms: how common are eating disorders in pregnancy?. Eur Eat Disord Rev. 2013;21(4):340–4. 10.1002/erv.2229 23495197

[7] Micali N, Simonoff E, Treasure J. Risk of major adverse perinatal outcomes in women with eating disorders. Br J Psychiatry. 2007;190:255–9. 10.1192/bjp.bp.106.020768 17329747

[8] Rickards AL, Kelly EA, Doyle LW, Callanan C. Cognition, academic progress, behavior and self-concept at 14 years of very low birth weight children. J Dev Behav Pediatr. 2001;22(1):11–8. 11265918 10.1097/00004703-200102000-00002

[9] Nomura Y, Wickramaratne PJ, Pilowsky DJ, Newcorn JH, Bruder-Costello B, Davey C, et al Low birth weight and risk of affective disorders and selected medical illness in offspring at high and low risk for depression. Compr Psychiatry. 2007;48(5):470–8. 10.1016/j.comppsych.2007.04.005 17707257 PMC2085442

[10] Lussana F, Painter RC, Ocke MC, Buller HR, Bossuyt PM, Roseboom TJ. Prenatal exposure to the Dutch famine is associated with a preference for fatty foods and a more atherogenic lipid profile. Am J Clin Nutr. 2008;88(6):1648–52. 10.3945/ajcn.2008.26140 19064527

[11] Palou M, Priego T, Sanchez J, Palou A, Pico C. Sexual dimorphism in the lasting effects of moderate caloric restriction during gestation on energy homeostasis in rats is related with fetal programming of insulin and leptin resistance. Nutr Metab (Lond). 2010;7:69,7075-7-69. 20796266 10.1186/1743-7075-7-69 PMC2939651

[12] Lukaszewski MA, Mayeur S, Fajardy I, Delahaye F, Dutriez-Casteloot I, Montel V, et al Maternal prenatal undernutrition programs adipose tissue gene expression in adult male rat offspring under high-fat diet. Am J Physiol Endocrinol Metab. 2011;301(3):E548–59. 10.1152/ajpendo.00011.2011 21712534

[13] Desai M, Gayle D, Babu J, Ross MG. Programmed obesity in intrauterine growth-restricted newborns: modulation by newborn nutrition. Am J Physiol Regul Integr Comp Physiol. 2005;288(1):R91–6. 10.1152/ajpregu.00340.2004 15297266

[14] Akitake Y, Katsuragi S, Hosokawa M, Mishima K, Ikeda T, Miyazato M, et al Moderate maternal food restriction in mice impairs physical growth, behavior, and neurodevelopment of offspring. Nutr Res. 2015;35(1):76–87. 10.1016/j.nutres.2014.10.014 25433908

[15] Suzuki M, Shibanuma M, Kimura S. Effect of severe maternal dietary restriction on growth and intra-abdominal adipose tissue weights in offspring rats. J Nutr Sci Vitaminol (Tokyo). 2010;56(5):293–8. 21228499 10.3177/jnsv.56.293

[16] Desai M, Babu J, Ross MG. Programmed metabolic syndrome: prenatal undernutrition and postweaning overnutrition. Am J Physiol Regul Integr Comp Physiol. 2007;293(6):R2306–14. 10.1152/ajpregu.00783.2006 17898113

[17] Aiken CE, Ozanne SE. Sex differences in developmental programming models. Reproduction. 2013;145(1):R1 10.1530/REP-11-0489 23081892

[18] McMillen IC, Schwartz J, Coulter CL, Edwards LJ. Early embryonic environment, the fetal pituitary-adrenal axis and the timing of parturition. Endocr Res. 2004;30(4):845–50. 15666835 10.1081/erc-200044106

[19] Zhang S, Morrison JL, Gill A, Rattanatray L, MacLaughlin SM, Kleemann D, et al Dietary restriction in the periconceptional period in normal-weight or obese ewes results in increased abundance of angiotensin-converting enzyme (ACE) and angiotensin type 1 receptor (AT1R) in the absence of changes in ACE or AT1R methylation in the adrenal of the offspring. Reproduction. 2013;146(5):443–54. 10.1530/REP-13-0219 24084173

[20] Levay EA, Paolini AG, Govic A, Hazi A, Penman J, Kent S. Anxiety-like behaviour in adult rats perinatally exposed to maternal calorie restriction. Behav Brain Res. 2008;191(2):164–72. 10.1016/j.bbr.2008.03.021 18453007

[21] Coupe B, Amarger V, Grit I, Benani A, Parnet P. Nutritional programming affects hypothalamic organization and early response to leptin. Endocrinology. 2010;151(2):702–13. 10.1210/en.2009-0893 20016030

[22] Delahaye F, Breton C, Risold P, Enache M, Dutriez-Casteloot I, Laborie C, et al Maternal perinatal undernutrition drastically reduces postnatal leptin surge and affects the development of arcuate nucleus proopiomelanocortin neurons in neonatal male rat pups. Endocrinology. 2008;149(2):470 10.1210/en.2007-1263 18006626

[23] Coupe B, Dutriez-Casteloot I, Breton C, Lefevre F, Mairesse J, Dickes-Coopman A, et al Perinatal undernutrition modifies cell proliferation and brain-derived neurotrophic factor levels during critical time-windows for hypothalamic and hippocampal development in the male rat. J Neuroendocrinol. 2009;21(1):40–8. 10.1111/j.1365-2826.2008.01806.x 19094092

[24] Madore C, Nadjar A, Delpech JC, Sere A, Aubert A, Portal C, et al Nutritional n-3 PUFAs deficiency during perinatal periods alters brain innate immune system and neuronal plasticity-associated genes. Brain Behav Immun. 2014;41:22–31. 10.1016/j.bbi.2014.03.021 24735929

[25] Di Marzo V, Goparaju SK, Wang L, Liu J, Batkai S, Jarai Z, et al Leptin-regulated endocannabinoids are involved in maintaining food intake. Nature. 2001;410(6830):822–5. 10.1038/35071088 11298451

[26] Keimpema E, Hokfelt T, Harkany T, Doherty P. The molecular interplay between endocannabinoid and neurotrophin signals in the nervous system and beyond. Eur J Neurosci 2014;39(3):334–43. 10.1111/ejn.12431 24494674

[27] Keimpema E, Calvigioni D, Harkany T. Endocannabinoid signals in the developmental programming of delayed-onset neuropsychiatric and metabolic illnesses. Biochem Soc Trans 2013;41(6):1569–76. 10.1042/BST20130117 24256256

[28] Cristino L, Becker T, Di Marzo V. Endocannabinoids and energy homeostasis: an update. Biofactors 2014;40(4):389–97. 10.1002/biof.1168 24752980

[29] Lutz B, Marsicano G, Maldonado R, Hillard CJ. The endocannabinoid system in guarding against fear, anxiety and stress. Nature reviews.Neuroscience. 2015;16(12):705–18. 10.1038/nrn4036 26585799 PMC5871913

[30] Lutz B. Endocannabinoid signals in the control of emotion. Curr Opin Pharmacol. 2009;9(1):46–52. 10.1016/j.coph.2008.12.001 19157983

[31] Tibirica E. The multiple functions of the endocannabinoid system: a focus on the regulation of food intake. Diabetol Metab Syndr. 2010;2:5,5996-2-5. 10.1186/1758-5996-2-5 20180990 PMC2832623

[32] Hansen HS, Artmann A. Endocannabinoids and nutrition. J Neuroendocrinol. 2008;20 Suppl 1:94–9. 18426507 10.1111/j.1365-2826.2008.01687.x

[33] Morgese MG, Trabace L. Maternal Malnutrition in the Etiopathogenesis of Psychiatric Diseases: Role of Polyunsaturated Fatty Acids. Brain Sci. 2016;6(3). 10.3390/brainsci6030024 PMC5039453 27472366

[34] Morgese MG, Tucci P, Mhillaj E, Bove M, Schiavone S, Trabace L, et al Lifelong Nutritional Omega-3 Deficiency Evokes Depressive-Like State Through Soluble Beta Amyloid. Mol Neurobiol. 2016. 10.1007/s12035-016-9809-2 PMC5355522 26924315

[35] Larrieu T, Madore C, Joffre C, Laye S. Nutritional n-3 polyunsaturated fatty acids deficiency alters cannabinoid receptor signaling pathway in the brain and associated anxiety-like behavior in mice. J Physiol Biochem. 2012;68(4):671–81. 10.1007/s13105-012-0179-6 22707188

[36] Lafourcade M, Larrieu T, Mato S, Duffaud A, Sepers M, Matias I, et al Nutritional omega-3 deficiency abolishes endocannabinoid-mediated neuronal functions. Nat Neurosci. 2011;14(3):345–50. 10.1038/nn.2736 21278728

[37] Hadley KB, Ryan AS, Forsyth S, Gautier S, Salem J, Norman. The Essentiality of Arachidonic Acid in Infant Development. Nutrients. 2016;8(4):216 10.3390/nu8040216 27077882 PMC4848685

[38] Maccarrone M, Guzman M, Mackie K, Doherty P, Harkany T. Programming of neural cells by (endo)cannabinoids: from physiological rules to emerging therapies. Nat Rev Neurosci. 2014;15(12):786–801. 10.1038/nrn3846 25409697 PMC4765324

[39] Antonelli T, Tomasini MC, Tattoli M, Cassano T, Tanganelli S, Finetti S, et al Prenatal exposure to the CB1 receptor agonist WIN 55,212–2 causes learning disruption associated with impaired cortical NMDA receptor function and emotional reactivity changes in rat offspring. Cereb Cortex. 2005;15(12):2013–20. 10.1093/cercor/bhi076 15788701

[40] Bernard C, Milh M, Morozov YM, Ben-Ari Y, Freund TF, Gozlan H. Altering cannabinoid signaling during development disrupts neuronal activity. Proc Natl Acad Sci U S A. 2005;102(26):9388–93. 10.1073/pnas.0409641102 15964987 PMC1166590

[41] de Salas-Quiroga A, Diaz-Alonso J, Garcia-Rincon D, Remmers F, Vega D, Gomez-Canas M, et al Prenatal exposure to cannabinoids evokes long-lasting functional alterations by targeting CB1 receptors on developing cortical neurons. Proc Natl Acad Sci U S A. 2015;112(44):13693–8. 10.1073/pnas.1514962112 26460022 PMC4640742

[42] Rodriguez de Fonseca F, Cebeira M, Fernandez-Ruiz JJ, Navarro M, Ramos JA. Effects of pre- and perinatal exposure to hashish extracts on the ontogeny of brain dopaminergic neurons. Neuroscience. 1991;43(2–3):713–23. 1922791 10.1016/0306-4522(91)90329-m

[43] Moreno M, Escuredo L, Munoz R, Rodriguez de Fonseca F, Navarro M. Long-term behavioural and neuroendocrine effects of perinatal activation or blockade of CB1 cannabinoid receptors. Behav Pharmacol. 2005;16(5–6):423–30. 16148447 10.1097/00008877-200509000-00015

[44] D'Asti E, Long H, Tremblay-Mercier J, Grajzer M, Cunnane SC, Di Marzo V, et al Maternal dietary fat determines metabolic profile and the magnitude of endocannabinoid inhibition of the stress response in neonatal rat offspring. Endocrinology. 2010;151(4):1685–94. 10.1210/en.2009-1092 20160134

[45] Matias I, Leonhardt M, Lesage J, De Petrocellis L, Dupouy JP, Vieau D, et al Effect of maternal under-nutrition on pup body weight and hypothalamic endocannabinoid levels. Cell Mol Life Sci. 2003;60(2):382–9. 12678501 10.1007/s000180300031 PMC11138768

[46] Berrendero F, Sepe N, Ramos JA, Di Marzo V, Fernandez-Ruiz JJ. Analysis of cannabinoid receptor binding and mRNA expression and endogenous cannabinoid contents in the developing rat brain during late gestation and early postnatal period. Synapse. 1999;33(3):181–91. 10.1002/(SICI)1098-2396(19990901)33:3<181::AID-SYN3>3.0.CO;2-R 10420166

[47] Ramírez-López MT, Vazquez M, Bindila L, Lomazzo E, Hofmann C, Blanco RN, et al Maternal Caloric Restriction Implemented during the Preconceptional and Pregnancy Period Alters Hypothalamic and Hippocampal Endocannabinoid Levels at Birth and Induces Overweight and Increased Adiposity at Adulthood in Male Rat Offspring. Front Behav Neurosci. 2016;10:208 10.3389/fnbeh.2016.00208 27847471 PMC5088205

[48] Soria-Gomez E, Bellocchio L, Reguero L, Lepousez G, Martin C, Bendahmane M, et al The endocannabinoid system controls food intake via olfactory processes. Nat Neurosci. 2014;17(3):407–15. 10.1038/nn.3647 24509429

[49] Bindila L, Lutz B. Extraction and Simultaneous Quantification of Endocannabinoids and Endocannabinoid-Like Lipids in Biological Tissues. Methods Mol Biol. 2016;1412:9–18. 10.1007/978-1-4939-3539-0_2 27245887

[50] Wenzel D, Matthey M, Bindila L, Lerner R, Lutz B, Zimmer A, et al Endocannabinoid anandamide mediates hypoxic pulmonary vasoconstriction. Proc Natl Acad Sci U S A. 2013;110(46):18710–5. 10.1073/pnas.1308130110 24167249 PMC3831960

[51] Ramírez-López MT, Vazquez M, Bindila L, Lomazzo E, Hofmann C, Blanco RN, et al Exposure to a Highly Caloric Palatable Diet During Pregestational and Gestational Periods Affects Hypothalamic and Hippocampal Endocannabinoid Levels at Birth and Induces Adiposity and Anxiety-Like Behaviors in Male Rat Offspring. Front Behav Neurosci. 2016;9:339 10.3389/fnbeh.2015.00339 26778987 PMC4701936

[52] Micali N, Treasure J, Simonoff E. Eating disorders symptoms in pregnancy: a longitudinal study of women with recent and past eating disorders and obesity. J Psychosom Res. 2007;63(3):297–303. 10.1016/j.jpsychores.2007.05.003 17719368

[53] Garcia AP, Palou M, Priego T, Sanchez J, Palou A, Pico C. Moderate caloric restriction during gestation results in lower arcuate nucleus NPY- and alphaMSH-neurons and impairs hypothalamic response to fed/fasting conditions in weaned rats. Diabetes Obes Metab. 2010;12(5):403–13. 10.1111/j.1463-1326.2009.01174.x 20415688

[54] Garcia AP, Palou M, Sanchez J, Priego T, Palou A, Pico C. Moderate caloric restriction during gestation in rats alters adipose tissue sympathetic innervation and later adiposity in offspring. PLoS One. 2011;6(2):e17313 10.1371/journal.pone.0017313 21364997 PMC3041800

[55] Ramírez-López MT, Arco R, Decara J, Vazquez M, Rivera P, Blanco RN, et al Long-Term Effects of Prenatal Exposure to Undernutrition on Cannabinoid Receptor-Related Behaviors: Sex and Tissue-Specific Alterations in the mRNA Expression of Cannabinoid Receptors and Lipid Metabolic Regulators. Front Behav Neurosci. 2016;10:241 10.3389/fnbeh.2016.00241 28082878 PMC5187359

[56] Cetin I, Alvino G, Cardellicchio M. Long chain fatty acids and dietary fats in fetal nutrition. J Physiol (Lond). 2009;587(14):3441–51. 19528253 10.1113/jphysiol.2009.173062 PMC2742273

[57] Amusquivar E, Herrera E. Influence of changes in dietary fatty acids during pregnancy on placental and fetal fatty acid profile in the rat. Biol Neonate. 2003;83(2):136–45. 12576758 10.1159/000067963

[58] Herrera E. Implications of Dietary Fatty Acids During Pregnancy on Placental, Fetal and Postnatal Development—A Review. Placenta. 2002;23, Supplement A(0):S9–S19. 11978055 10.1053/plac.2002.0771

[59] Gallou-Kabani C, Gabory A, Tost J, Karimi M, Mayeur S, Lesage J, et al Sex- and diet-specific changes of imprinted gene expression and DNA methylation in mouse placenta under a high-fat diet. PLoS One. 2010;5(12):e14398 10.1371/journal.pone.0014398 21200436 PMC3006175

[60] Gabory A, Ferry L, Fajardy I, Jouneau L, Gothie JD, Vige A, et al Maternal diets trigger sex-specific divergent trajectories of gene expression and epigenetic systems in mouse placenta. PLoS One. 2012;7(11):e47986 10.1371/journal.pone.0047986 23144842 PMC3489896

[61] Bieswal F, Ahn MT, Reusens B, Holvoet P, Raes M, Rees WD, et al The importance of catch-up growth after early malnutrition for the programming of obesity in male rat. Obesity (Silver Spring). 2006;14(8):1330–43. 16988075 10.1038/oby.2006.151

[62] Williams CM, Kirkham TC. Anandamide induces overeating: mediation by central cannabinoid (CB1) receptors. Psychopharmacology (Berl). 1999;143(3):315–7. 10353436 10.1007/s002130050953

[63] Jamshidi N, Taylor DA. Anandamide administration into the ventromedial hypothalamus stimulates appetite in rats. Br J Pharmacol. 2001;134(6):1151–4. 10.1038/sj.bjp.0704379 11704633 PMC1573067

[64] De Fonseca FR, Navarro M, Gomez R, Escuredo L, Nava F, Fu J, et al An anorexic lipid mediator regulated by feeding. Nature-London. 2001:209–11. 10.1038/35102582 11700558

[65] Hoareau L, Roche R. Palmitoylethanolamide, adipocytes and obesity-related inflammatory states. Drug Discovery Today: Disease Mechanisms. 2010;7(3–4):e205–12.

[66] Mattace Raso G, Santoro A, Russo R, Simeoli R, Paciello O, Di Carlo C, et al Palmitoylethanolamide prevents metabolic alterations and restores leptin sensitivity in ovariectomized rats. Endocrinology. 2014;155(4):1291 10.1210/en.2013-1823 24428531 PMC5393333

[67] Rana JS, Nieuwdorp M, Jukema JW, Kastelein JJP. Cardiovascular metabolic syndrome–an interplay of, obesity, inflammation, diabetes and coronary heart disease. Diabetes, Obesity and Metabolism. 2007;9(3):218–32. 10.1111/j.1463-1326.2006.00594.x 17391148

[68] Bolton JL, Bilbo SD. Developmental programming of brain and behavior by perinatal diet: focus on inflammatory mechanisms. Dialogues Clin Neurosci. 2014;16(3):307–20. 25364282 10.31887/DCNS.2014.16.3/jbolton PMC4214174

[69] Marques AH, Bjorke-Monsen AL, Teixeira AL, Silverman MN. Maternal stress, nutrition and physical activity: Impact on immune function, CNS development and psychopathology. Brain Res. 2015;1617:28–46. 10.1016/j.brainres.2014.10.051 25451133

[70] Artmann A, Petersen G, Hellgren LI, Boberg J, Skonberg C, Nellemann C, et al Influence of dietary fatty acids on endocannabinoid and N-acylethanolamine levels in rat brain, liver and small intestine. Biochim Biophys Acta. 2008;1781(4):200–12. 10.1016/j.bbalip.2008.01.006 18316044

[71] Hansen HS. Effect of diet on tissue levels of palmitoylethanolamide. CNS Neurol Disord Drug Targets. 2013;12(1):17–25. 23394522 10.2174/1871527311312010006

[72] Pu S, Eck P, Jenkins DJ, Connelly PW, Lamarche B, Kris-Etherton PM, et al Interactions between dietary oil treatments and genetic variants modulate fatty acid ethanolamides in plasma and body weight composition. Br J Nutr. 2016;115(6):1012–23. 10.1017/S0007114515005425 26806592

[73] Rivera P, Luque-Rojas MJ, Pastor A, Blanco E, Pavón FJ, Serrano A, et al Diet-dependent modulation of hippocampal expression of endocannabinoid signaling-related proteins in cannabinoid antagonist-treated obese rats. Eur J Neurosci. 2012: 37(1):105–17. 10.1111/ejn.12012 23033907

[74] Fezza F, Bari M, Florio R, Talamonti E, Feole M, Maccarrone M. Endocannabinoids, related compounds and their metabolic routes. Molecules. 2014;19(11):17078–106. 10.3390/molecules191117078 25347455 PMC6271436

[75] Jenniches I, Ternes S, Albayram O, Otte DM, Bach K, Bindila L, et al Anxiety, Stress, and Fear Response in Mice with Reduced Endocannabinoid Levels. Biol Psychiatry. 2015;79(10):858–68. 10.1016/j.biopsych.2015.03.033 25981172

[76] Guggenhuber S, Romo-Parra H, Bindila L, Leschik J, Lomazzo E, Remmers F, et al Impaired 2-AG Signaling in Hippocampal Glutamatergic Neurons: Aggravation of Anxiety-Like Behavior and Unaltered Seizure Susceptibility. Int J Neuropsychopharmacol. 2015;19(2):pii: pyv091. 10.1093/ijnp/pyv091 PMC4772822 26232789

[77] Campos AC, Ferreira FR, Guimarães FS, Lemos JI. Facilitation of endocannabinoid effects in the ventral hippocampus modulates anxiety-like behaviors depending on previous stress experience. Neuroscience. 2010;167(2):238–46. 10.1016/j.neuroscience.2010.01.062 20167262

[78] Avraham Y, Saidian M, Burston JJ, Mevorach R, Vorobiev L, Magen I, et al Fish oil promotes survival and protects against cognitive decline in severely undernourished mice by normalizing satiety signals. J Nutr Biochem. 2011;22(8):766–76. 10.1016/j.jnutbio.2010.07.001 21109417 PMC3117120

[79] Clarke DJ, Stuart J, McGregor IS, Arnold JC. Endocannabinoid dysregulation in cognitive and stress-related brain regions in the Nrg1 mouse model of schizophrenia. Prog Neuropsychopharmacol Biol Psychiatry 2017;72:9–15. 10.1016/j.pnpbp.2016.08.006 27521758

[80] Almeida SS, Tonkiss J, Galler JR. Prenatal protein malnutrition affects exploratory behavior of female rats in the elevated plus-maze test. Physiol Behav. 1996;60(2):675–80. 8840934 10.1016/s0031-9384(96)80047-3

[81] Reyes-Castro LA, Rodriguez JS, Charco R, Bautista CJ, Larrea F, Nathanielsz PW, et al Maternal protein restriction in the rat during pregnancy and/or lactation alters cognitive and anxiety behaviors of female offspring. Int J Dev Neurosci. 2012;30(1):39–45. 10.1016/j.ijdevneu.2011.10.002 22023958

[82] Reyes-Castro LA, Rodriguez JS, Rodriguez-Gonzalez GL, Chavira R, Bautista CJ, McDonald TJ, et al Pre- and/or postnatal protein restriction developmentally programs affect and risk assessment behaviors in adult male rats. Behav Brain Res. 2012;227(2):324–9. 10.1016/j.bbr.2011.06.008 21704656

[83] de Souza MA, Szawka RE, Centenaro LA, Diehl LA, Lucion AB. Prenatal stress produces sex differences in nest odor preference. Physiol Behav. 2012;105(3):850–5. 10.1016/j.physbeh.2011.10.012 22037198

[84] Watanabe S, Doshi M, Hamazaki T. n-3 Polyunsaturated fatty acid (PUFA) deficiency elevates and n-3 PUFA enrichment reduces brain 2-arachidonoylglycerol level in mice. Prostaglandins Leukot Essent Fatty Acids. 2003;69(1):51–9. 12878451 10.1016/s0952-3278(03)00056-5

[85] Dyall SC, Mandhair HK, Fincham RE, Kerr DM, Roche M, Molina-Holgado F. Distinctive effects of eicosapentaenoic and docosahexaenoic acids in regulating neural stem cell fate are mediated via endocannabinoid signalling pathways. Neuropharmacology. 2016;107:387–95. 10.1016/j.neuropharm.2016.03.055 27044662

